# Ultraviolet B, melanin and mitochondrial DNA: Photo-damage in human epidermal keratinocytes and melanocytes modulated by alpha-melanocyte-stimulating hormone

**DOI:** 10.12688/f1000research.8582.1

**Published:** 2016-05-12

**Authors:** Markus Böhm, Helene Z. Hill

**Affiliations:** 1 Department of Dermatology, University of Münster, Münster, Germany; 2 Department of Radiology, Rutgers New Jersey Medical School, Newark, NJ, USA

**Keywords:** Melanin, alpha-melanocyte stimulating hormone, UVB, ) melanocytes, keratinocytes, mitochondrial DNA, mitochondrial DNA deletions, polymerase chain reaction

## Abstract

Alpha-melanocyte-stimulating hormone (alpha-MSH) increases melanogenesis and protects from UV-induced DNA damage. However, its effect on mitochondrial DNA (mtDNA) damage is unknown. We have addressed this issue in a pilot study using human epidermal keratinocytes and melanocytes incubated with alpha-MSH and irradiated with UVB. Real-time touchdown PCR was used to quantify total and deleted mtDNA. The deletion detected encompassed the common deletion but was more sensitive to detection. There were 4.4 times more mtDNA copies in keratinocytes than in melanocytes. Irradiation alone did not affect copy numbers. Alpha-MSH slightly increased copy numbers in both cell types in the absence of UVB and caused a similar small decrease in copy number with dose in both cell types. Deleted copies were nearly twice as frequent in keratinocytes as in melanocytes. Alpha-MSH reduced the frequency of deleted copies by half in keratinocytes but not in melanocytes. UVB dose dependently led to an increase in the deleted copy number in alpha-MSH-treated melanocytes. UVB irradiation had little effect on deleted copy number in alpha-MSH-treated keratinocytes. In summary, alpha-MSH enhances mtDNA damage in melanocytes presumably by increased melanogenesis, while α-MSH is protective in keratinocytes, the more so in the absence of irradiation.

## Introduction

Melanin is found in the cell cytoplasm as are the mitochondria. Although the principal role of melanin is photoprotection, the pigment is well known to emit melanin free radicals and act as a photosensitizing agent ^1, 2^. These studies were undertaken to determine the effect of ultraviolet B irradiation (UVB) on the induction of reactive oxygen species damage of mitochondrial DNA (mtDNA) in cultures of pigment-induced human epidermal melanocytes compared to human epidermal keratinocytes. Deletions in mitochondrial DNA are the hallmark of reactive oxygen species damage.

## Materials and methods

### Cells and cell culture conditions

Normal human neonatal epidermal melanocytes were purchased from Tebu-bio (Portland, OR) (catalog # 104-05n 5E5 Cells) and normal human neonatal epidermal keratinocytes were obtained from PromoCell (Heidelberg, Germany) (catalog # C-12007). Melanocytes and keratinocytes were routinely cultured as reported previously ^3^. Melanocytes were maintained in MGM-M2 medium plus all supplements (Cascade Biologics, Portland, OR; M-254-500 plus S-002-5) while keratinocytes were cultured in KBM-2 medium with all supplements (PromoCell; C-20111). 500,000–700,000 cells were seeded into 6 cm diameter culture dishes. On the following day cells were deprived for 48 hrs from bovine pituitary extract followed by pre-incubation with 10 ^-6^ M α-MSH (α-melanocyte-stimulating hormone) (Calbiochem, Schwalbach, Germany) for 6 hrs at 37°C.

### Irradiation

UVB treatment was performed with an irradiation bank consisting of six fluorescent bulbs (TL12, Philips) which emit most of their energy within the UVB range with an emission peak of 313 nm. Cells were irradiated through phosphate-buffered saline at 5, 10 and 15 mJ/cm ^2^ followed by incubation with experimental medium (5 ml) with and without α-MSH (10 ^-6^ M) for 24 hrs.

### DNA extraction and purification

DNA was extracted using the Epicentre kit from Biozym (Hess. Oldendorf, Germany; MCD85201). DNA was finally dissolved in 10 mM Tris buffer with 0.1 mM EDTA.

### Restriction and repurification

Five micrograms of each purified DNA sample were incubated with 10 units of AflIII (New England BioLabs, Ipswich, MA) for 1 hour at 37°C according to the manufacturer’s directions. The reaction mixture was repurified on spin columns according to the manufacturer’s directions (DNA Clean and Concentrator columns, Zymo Research Corporation, Orange, CA) and diluted appropriately in 10 mM Tris pH 8.5.

### PCR

The Roche LightCycler-2 (Roche Applied Science, Indianapolis, IN) was used throughout these studies. Total mitochondrial genomes were quantified using primers HSSN1307 and HSAS 1433 ^4^ (5’GTACCCACGTAAAGACGTTAGG3’ and 5’TACTGCTAAATCCACC-TTCG3’ respectively) and probe 5’FAM-CCCATGAGGCAAGAAATT-BHQ1-3’ ^3^. Each capillary contained 250 pg DNA, 300 nM each primer, 200 nM probe, 0.01 μL uracil-DNAglycosylase, heat-labile (Roche) (UNG) and 1X LightCycler TaqMan Master Mix in a total volume of 5 μL. The Master Mix contains dUTP in place of dTTP in order for the amplified product to contain U in place of T. Pre-incubation of the PCR mixture with UNG allows for the degradation of any carryover contaminants from earlier reactions. The cycling protocol called for 10 min at 35°C for UNG digestion, followed by 10 min at 95°C to inactivate UNG and activate the Taq polymerase; up to 40 cycles of 95°C 10 sec for denaturation, 60°C 20 sec for annealing, 72°C 10 sec for polymerization and acquisition. Each run also contained two capillaries of 1 × 10 ^5^ copies of the plasmid pKW into which the Total amplicon had been inserted ^4^.

Primers for the deletion were HSSN8416 and HSAS8542 (5’-CCTTACACTATTCCT-CATCACC-3’ and 5’-TGTGGTCTTTGGAGTAGAAACC-3’ respectively) ^3^. The probe was 5’-6FAM-TGGCAGCCTAGCATTAGCAGTT-BHQ1-3’) ^4^. Each capillary contained 10 ng sample DNA, primers, probe, UNG and Master Mix as in the Total reactions. Glycosylation and denaturation were as for the Total PCR. Amplification was initiated with 5 cycles of Touchdown PCR: cycle 1: 95°C for 10 sec, 65°C for 5 sec, dropping 1 degree per cycle down to 60°C followed by no more than 55 cycles of 95°C for 10 sec, 60°C for 15 sec. Results with touchdown PCR were more reproducible and took fewer cycles to reach the crossing point ^5^. The touchdown amplicon was sequenced in the Molecular Resource Facility at the NJ Medical School.

The results shown are averages of three separate experiments. The proprietary software accompanying the Roche LightCycler was used to determine the crossing point of each sample. The crossing points of the samples were compared to crossing points of known copy numbers of standards in order to calculate sample copy numbers. In each experiment, total copies were determined in triplicate and deleted copies were determined in quintuplicate. Please see Dataset 1.

## Results

Raw data for ‘Ultraviolet B, melanin and mitochondrial DNA: Photo-damage in human epidermal keratinocytes and melanocytes modulated by alpha-melanocyte-stimulating hormone’, Bohm and Hill 2016 Click here for additional data file. Copyright: © 2016 Böhm M and Hill HZ 2016 Data associated with the article are available under the terms of the Creative Commons Zero "No rights reserved" data waiver (CC0 1.0 Public domain dedication).

### Sequence of the touchdown amplicon

Gel analysis determined that the touchdown amplicon was smaller than that of the common deletion (CD): less than 100 bp versus 127 bp. The CD is 4977 bp and spans bp 8470 to bp 13447. The touchdown deletion (TD) spans 5021 bp from bp 8433 to bp 13454. The CD is contained within the TD. Just as the CD is based on repeat sequences, in this case, of 13 bp, ACCTCCCTCACCA, the TD is based on the 5 bp repeat TCACC. Note that the 5 bp repeat is actually contained in the 13 bp repeat and the 3’ ends of both deletions coincide. The 5’ ends of the two deletions are 37 bp apart.

**Figure 1.**
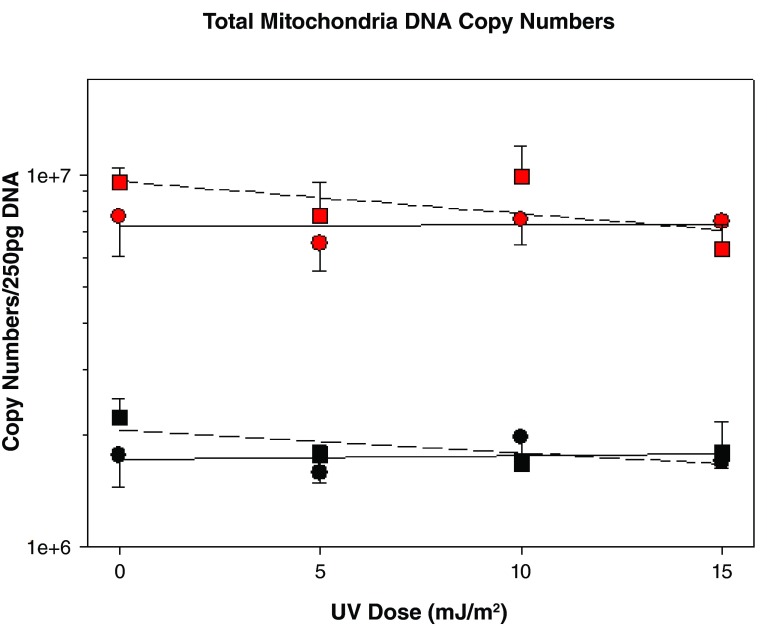
Total mtDNA copies as a function of UVB dose. Black symbols: melanocytes, red symbols: keratinocytes. Circles: no α-MSH; squares: + α-MSH.

Figure 1 shows the effect of UVB irradiation on total mitochondrial genome copy numbers in melanocytes and keratinocytes that have been treated with α-MSH, compared to untreated (no α-MSH) controls. In the absence of any α-MSH, keratinocytes have 4.4 times more mtDNA copies than melanocytes. Copy numbers of mtDNA from non-α-MSH treated cells of both cell types are unaffected by UVB exposure in this dose range. α-MSH causes a small increase in copy number in both cell types in the absence of irradiation and a parallel decline with dose.

**Figure 2.**
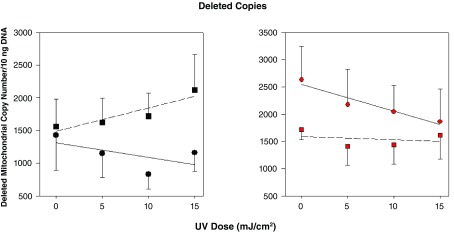
Deleted copies in melanocytes and keratinocytes as a function of UVB dose. Black symbols: melanocytes, red symbols: keratinocytes. Circles: no α-MSH; squares: + α-MSH.

Figure 2 shows the effect of UVB irradiation on deleted copy numbers in melanocytes and keratinocytes that have been exposed to α-MSH compared to non-α-MSH treated controls. In the absence of any α-MSH treatment, deleted copies are about half as frequent in melanocytes than in keratinocytes. In the absence of α-MSH treatment, deleted copies decline slightly with increasing radiation dose in both types of cell. This is more likely due to slower replication of the deleted genomes than to their direct loss.

In melanocytes pre-treated with α-MSH, there is a marked increase in the frequency of deleted genomes with increasing UVB dose. This is likely to be due to the production of reactive oxygen species from UVB interaction with the melanin pigment or with melanin precursors. α-MSH actually causes a decrease in deleted copies in unexposed keratinocytes suggesting that, after exposure deleted copies replicate more slowly or that some protective agent has been induced. This effect is lessened as the UVB dose increases.

## Discussion

The pigment melanin is widely distributed in the animal world and has multiple functions. In the skin, it is both photoprotective ^6– 8^ and photosensitizing ^9– 13^. The quixotic nature of melanin has been the subject of a number of studies and reviews ^2, 14– 18^.

α-MSH is a key melanotropic factor induced by UVB irradiation in keratinocytes to turn on melanogenesis. Of note, we and others have shown that α-MSH is capable of reducing UVB-mediated DNA damage in both human melanocytes and keratinocytes independent from its melanin-inducing effect ^19– 21^. Moreover, we recently found that α-MSH has indirect anti-oxidative effects on human melanocytes and keratinocytes since it upregulates expression of the transcription factor Nrf2, a master regulator of phase II detoxifying enzymes ^3^. Thus, we speculated that in both cell types α-MSH may also protect from some UVB-induced oxidative effects on mtDNA. The CD in mtDNA is a hallmark for oxidative stress. Arck *et al.* reported more CDs in graying hair follicles than in unpigmented follicles ^22^, suggesting that the pigment was responsible for the effect. Our findings in fact suggest that α-MSH-induced melanin synthesis acts as a photosensitizer for mtDNA damage in the form of the TD after irradiation with UVB in the MED (minimal erythema dose) range. The presence of melanin and/or its precursors in the cytoplasm may indeed produce oxidative damage in mtDNA which is enhanced by solar irradiation, thus altering the “ *milieu intérieur*” and possibly leading to altered mitochondrial function. In this context, it should be very interesting to determine the direct influence of α-MSH alone or in combination with UVB on mitochondrial metabolism and biogenesis. In addition, investigation on the impact of signaling-deficient *MC1R* alleles on mtDNA damage in melanocytes treated with α-MSH and UVB may disclose unexpected clues in the pathogenesis of cutaneous melanoma.

Recently, Premi *et al.* ^23^ irradiated mouse and human pigmented and non-pigmented cells with UVA and measured the production of cyclobutane pyrimidine dimers (CPDs) in total cellular DNA. They found that CPDs continued to increase in the pigmented cells, but not in the non-pigmented cells, for as long as 3 hours after irradiation, after which they declined due to repair. In our study, deletions were measured at only a single time point so there is no way of determining the dynamics of formation. However, once formed, they would persist unrepaired. It would be of interest to study the time-dependent effects of UVB and UVA on induction of mitochondrial deletions in unpigmented, pigmenting and pigmented cells, more especially since Boulton and Birch-Machin recently found that pigment is protective against reactive oxygen generation by complex II ^24^. Another demonstration, perhaps, of the dual nature of melanin as a two-edged sword ^15^. Function is protected while genetic information is damaged.

## Conclusion

There are about 2.4 times more deleted copies relative to total copies in melanocytes compared to keratinocytes. UVB increases the ratio in the presence of α-MSH in melanocytes while it remains relatively stable in keratinocytes. This suggests that α-MSH causes a destabilization of the free-radical balance in melanocytes but not in keratinocytes. This effect may be the result of the increase in melanin precursors and/or pigment by the presence of α-MSH.

## Data availability

The data referenced by this article are under copyright with the following copyright statement: Copyright: © 2016 Böhm M and Hill HZ

Data associated with the article are available under the terms of the Creative Commons Zero "No rights reserved" data waiver (CC0 1.0 Public domain dedication). 

*F1000Research*: Dataset 1. Raw data for ‘Ultraviolet B, melanin and mitochondrial DNA: Photo-damage in human epidermal keratinocytes and melanocytes modulated by alpha-melanocyte-stimulating hormone’, Böhm and Hill 2016, 10.5256/f1000research.8582.d121454 ^25^
