# Supplementary material for: Ultraviolet B, melanin and mitochondrial DNA: Photo-damage in human epidermal keratinocytes and melanocytes modulated by alpha-melanocyte-stimulating hormone
Source: F1000Res. 2016 May 12;5:881. [Version 1] doi: 10.12688/f1000research.8582.1 (PMC4890311; doi:10.12688/f1000research.8582.1)
Supplement: Peer review history

# Peer review: Referee response for version 1

**DOI:** 10.5256/f1000research.9236.r14092

**Published:** 2016-06-01

**Authors:** Jose Carlos Garcia-Borron

**Affiliations:** 1 Department of Biochemistry, Molecular Biology and Immunology, School of Medicine, University of Murcia, Murcia, Spain

This report from Markus Böhm and Helene Hill describes a study on the mitochondrial DNA damaging effect of ultraviolet B (UVB) radiation in keratinocytes and melanocytes, in the presence or absence of the melanotropic peptide hormone alphaMSH. It is known that binding of this melanocortin to its cognate receptor expressed in melanocytes, the MC1R, triggers a series of pleiotropic responses notably including activation of the melanogenic pathway and induction of antioxidant enzymes as well as DNA repair pathways. it is also known that MC1R is expressed in many nonmelanocytic cell types including keratinocytes. In fact, work in M Böhm’s lab has been crucial to the current view of alphaMSH/MC1R signaling as a key photoprotective process, and has led the field of extramelanocytic actions of MC1R. The contributions of HZ Hill have been also important to appreciate the dual character of melanins, with photoprotective roles but also endowed with a photosensitizing potential. Accordingly, the work is presented by leading experts in the field.

 Whereas the role of alphaMSH in mediating protection of nuclear DNA against UVB is well established, not much is known about a possible action on mitochondrial DNA. Therefore, the paper has the merit to address a still unexplored question. The authors report a higher frequency of damaged mitochondrial DNA in control keratinocytes not exposed to UVB, compared with control melanocytes. Rather counterintuitively, alphaMSH apparently protects mitochondrial DNA against UVB-induced damage in keratinocytes, whereas it promotes an increase in the number of damaged copies in melanocytes. Since the hormone stimulates melanogenesis in melanocytes but not in keratinocytes, this suggests that the melanin polymer or some unidentified melanogenic intermediates might in fact act as photosensitizers, in keeping with the two-edged sword character of melanin emphasized by previous contributions from HZ Hill.

 The experiments reported here are well described and most likely well conducted, and I have not detected any overinterpretation of the results. One caveat that leaves room for improvement is the absence of a statistical analysis of the small differences between the different experimental conditions.

 A major strength of this short report is that it raises new questions that are likely to be addressed in the near future. Do eumelanin-containing melanocytes from dark-skinned donors display the same responses to UVB in terms of mitochondrial DNA damage as feomelanogenic melanocytes from individuals with red hair color? Do melanocytes expressing the consensus MC1R respond to alphaMSH comparably to melanocytes expressing variant MC1R? Is there a differential effect of alphaMSH in melanocytes challenged with UVB in the presence of inhibitors of the rate-limiting melanogenic enzyme tyrosinase, or for that matter in albino melanocytes? And what is the impact of UVB and alphaMSH signaling on the performance of oxidative phosphorylation/electron transport chain as a main source of DNA-damaging reactive oxygen species? Some of these questions are mentioned in the short but well focused discussion, and their answer will contribute to a better understanding of the complex role of pigment production in the homeostasis of UVB-irradiated human skin.

I have read this submission. I believe that I have an appropriate level of expertise to confirm that it is of an acceptable scientific standard.

# Peer review: Referee response for version 1

**DOI:** 10.5256/f1000research.9236.r13934

**Published:** 2016-05-23

**Authors:** Andrzej Slominski

**Affiliations:** 1 Department of Dermatology , VA Medical Center, University of Alabama at Birmingham, Birmingham, AL, USA

This is well performed study by experts in the field. The methodology and data collection are sound and data properly discussed.

 I believe that that careful readers would like to have more extended list of references plus appropriate comments in the introduction and discussion.

 For example on the capability of the skin to express POMC and its processing to MSH, ACTH and endorphin peptides ^1^, on hormonal and nutritional regulation of melanin pigmentation with regulatory consequences, ^2^^,^^3^ and double-edge sword role of melanin in melanoma ^4^.

I have read this submission. I believe that I have an appropriate level of expertise to confirm that it is of an acceptable scientific standard.

## References

[1] SlominskiAWortsmanJLugerTPausRSolomonS: Corticotropin releasing hormone and proopiomelanocortin involvement in the cutaneous response to stress. *Physiol Rev*.2000;80(3) :979-1020 1089342910.1152/physrev.2000.80.3.979

[2] SlominskiATobinDJShibaharaSWortsmanJ: Melanin pigmentation in mammalian skin and its hormonal regulation. *Physiol Rev*.2004;84(4) : 10.1152/physrev.00044.2003 1155-228 10.1152/physrev.00044.2003 15383650

[3] SlominskiAZmijewskiMAPawelekJ: L-tyrosine and L-dihydroxyphenylalanine as hormone-like regulators of melanocyte functions. *Pigment Cell Melanoma Res*.2012;25(1) : 10.1111/j.1755-148X.2011.00898.x 14-27 10.1111/j.1755-148X.2011.00898.x 21834848PMC3242935

[4] SlominskiRMZmijewskiMASlominskiAT: The role of melanin pigment in melanoma. *Exp Dermatol*.2015;24(4) : 10.1111/exd.12618 258-9 10.1111/exd.12618 25496715PMC4450257
